# Supplementary figures and images for: Comprehensive Functional Annotation of Metagenomes and Microbial Genomes Using a Deep Learning-Based Method
Source: mSystems. 2023 Mar 7;8(2):e01178-22. doi: 10.1128/msystems.01178-22 (PMC10134832; doi:10.1128/msystems.01178-22)

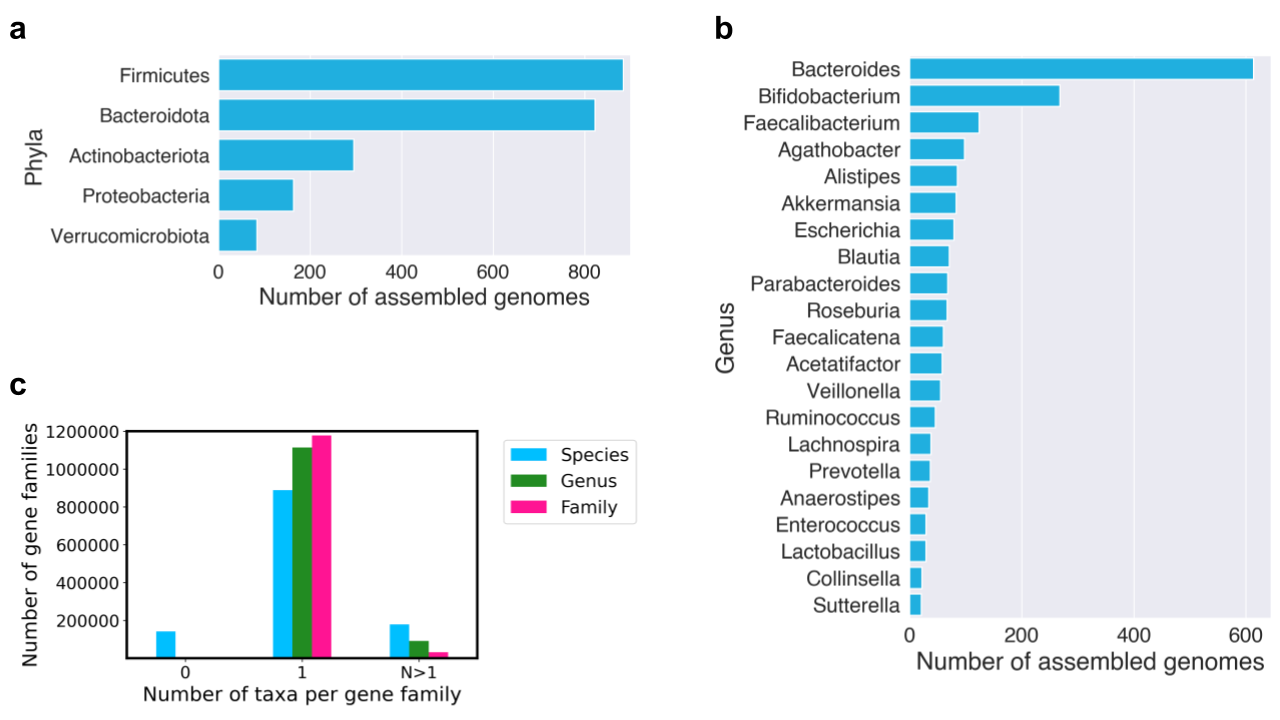

Supplement: FIG S1 [file msystems.01178-22-s0001.tif]

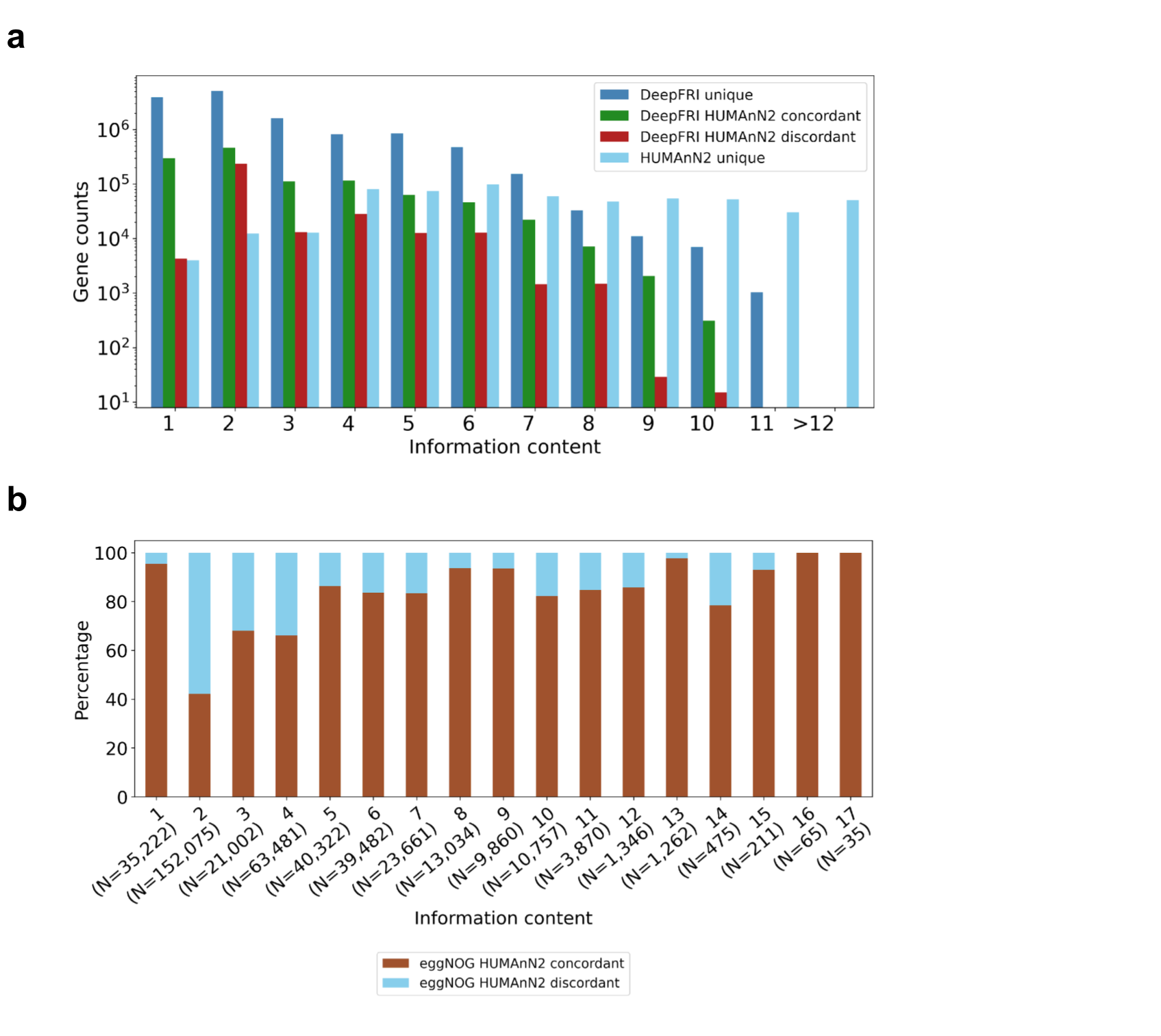

Supplement: FIG S2 [file msystems.01178-22-s0002.tif]

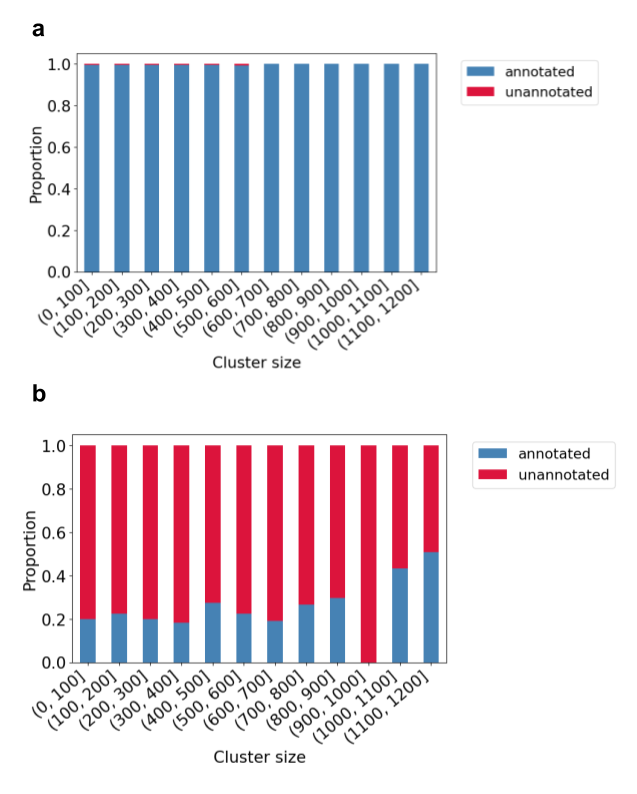

Supplement: FIG S3 [file msystems.01178-22-s0003.tif]

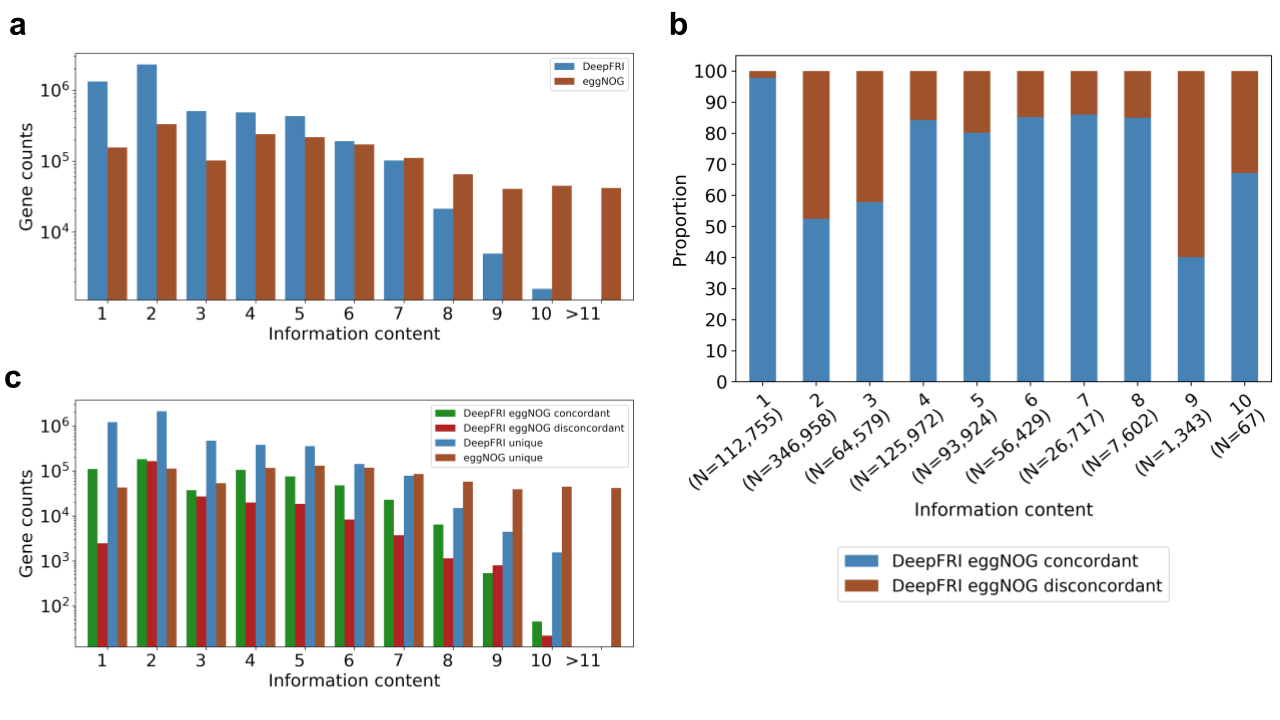

Supplement: FIG S4 [file msystems.01178-22-s0004.tif]

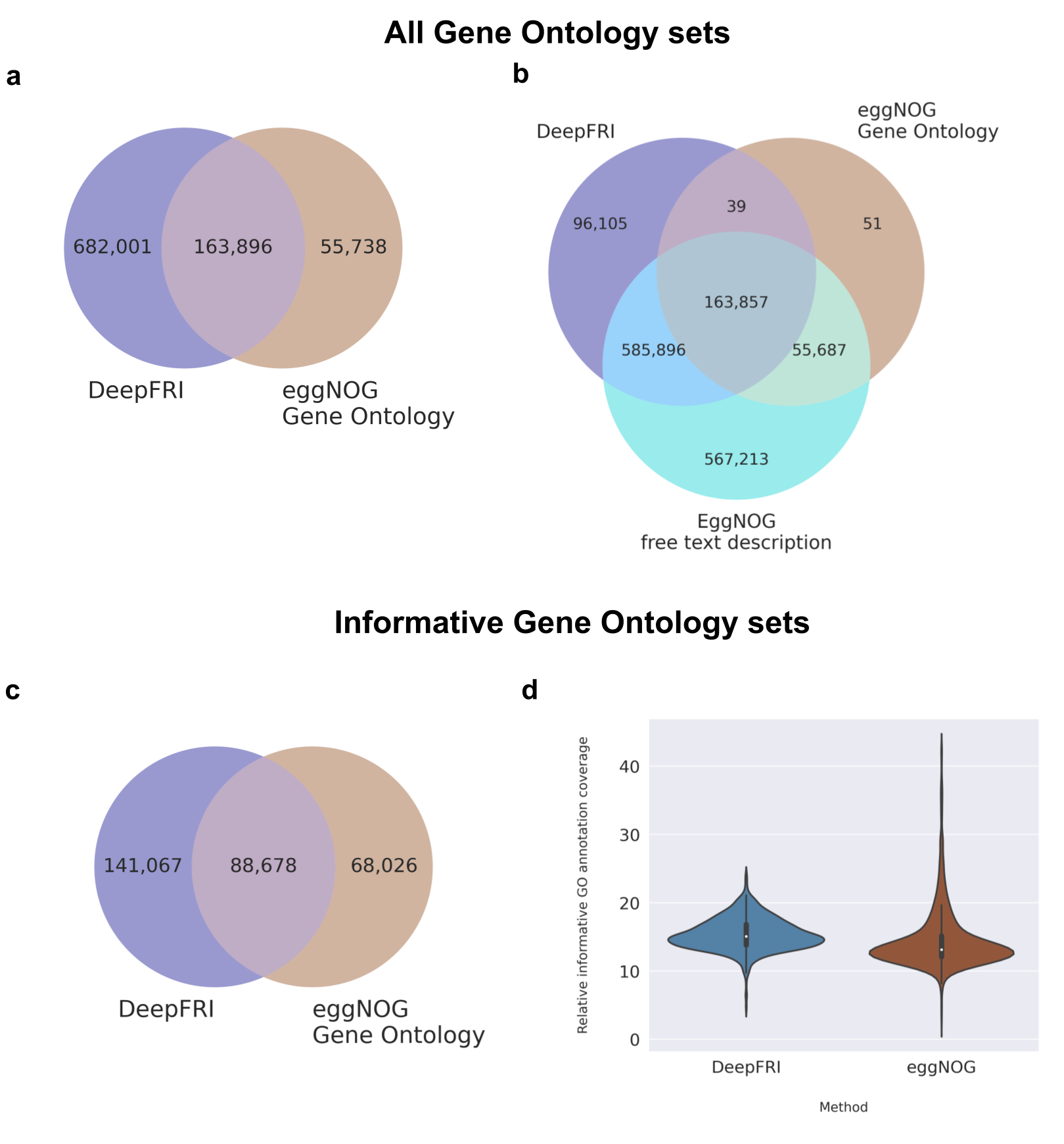

Supplement: FIG S5 [file msystems.01178-22-s0005.tif]

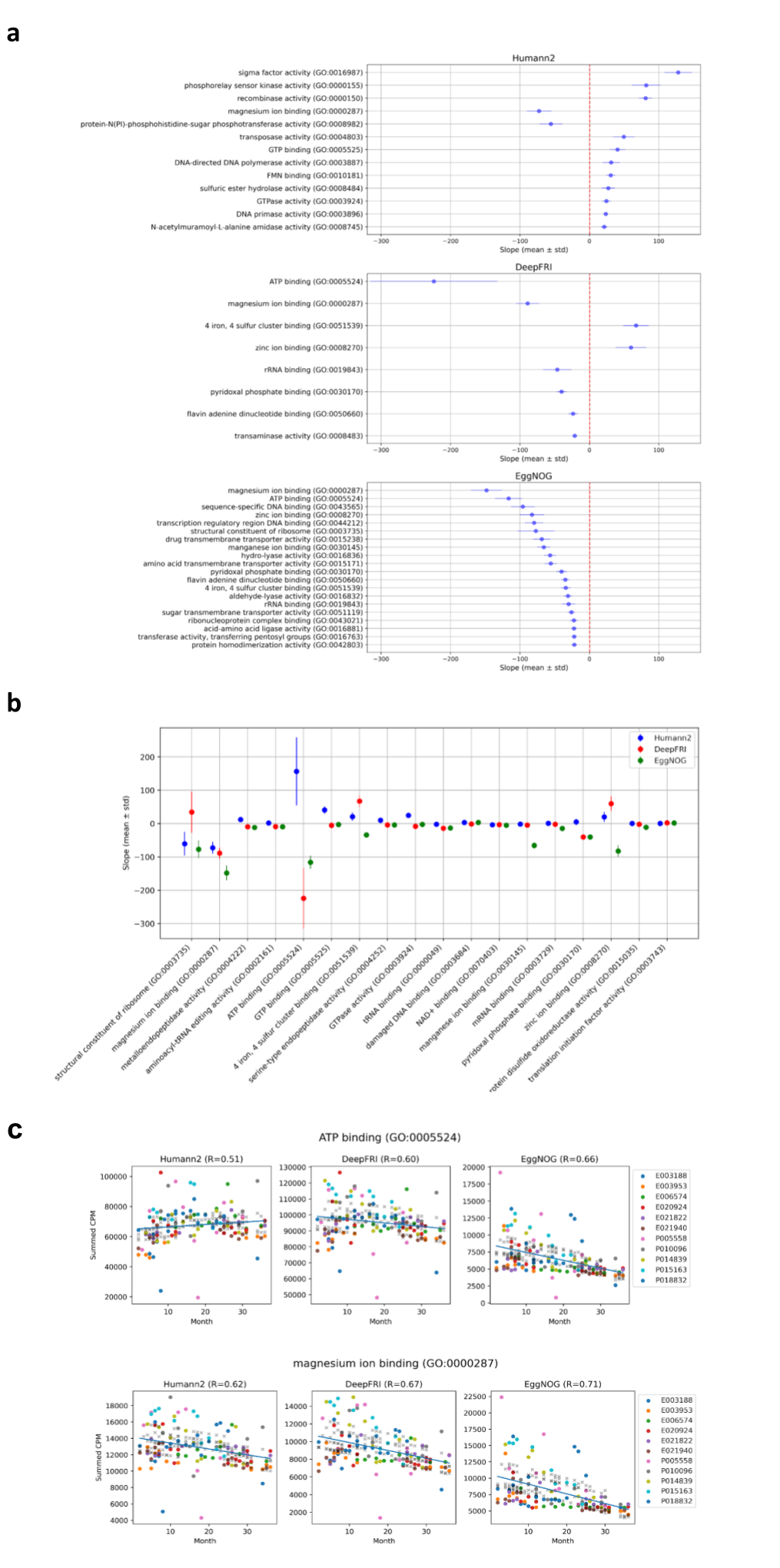

Supplement: FIG S6 [file msystems.01178-22-s0006.tif]

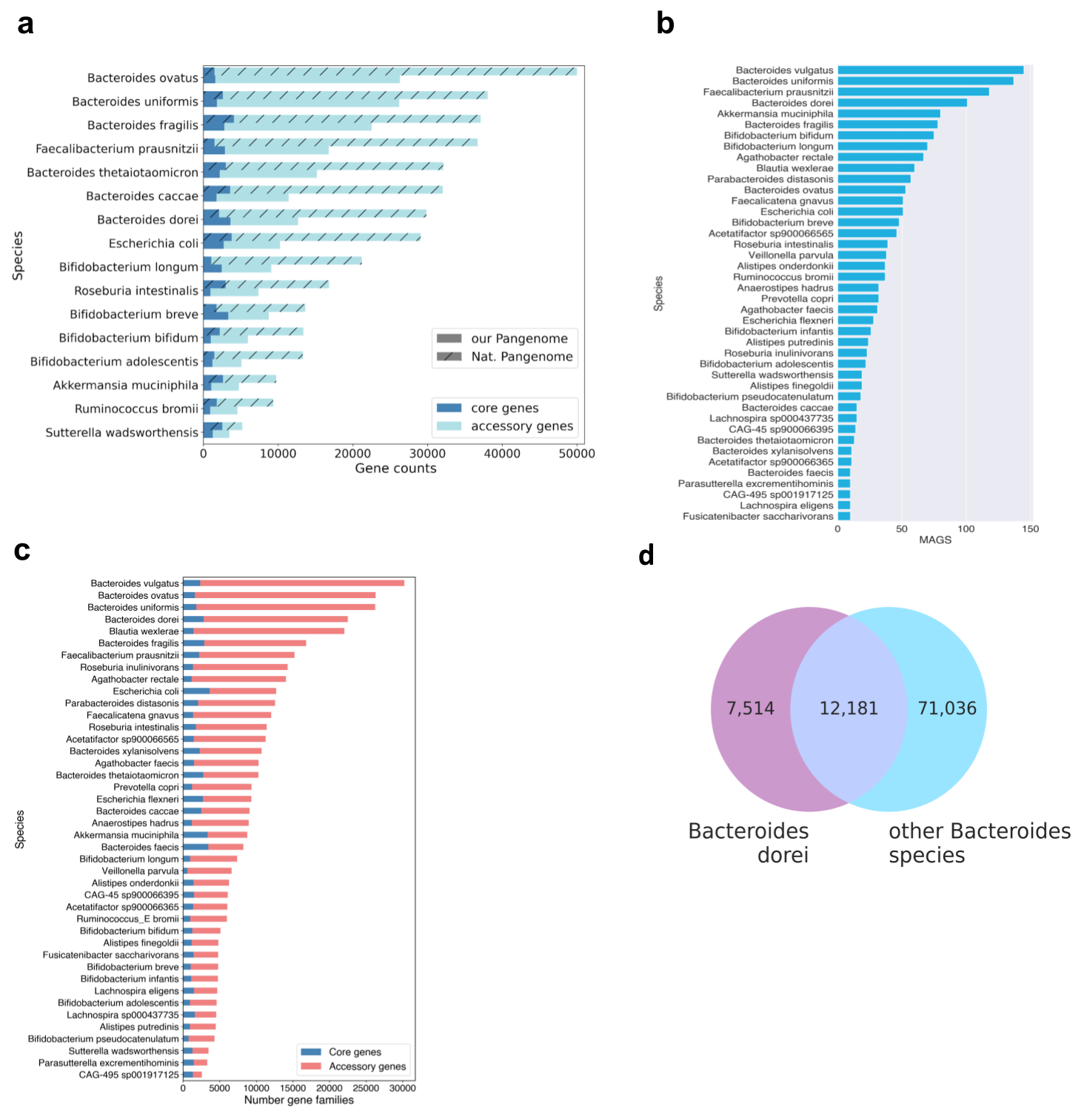

Supplement: FIG S7 [file msystems.01178-22-s0007.tif]

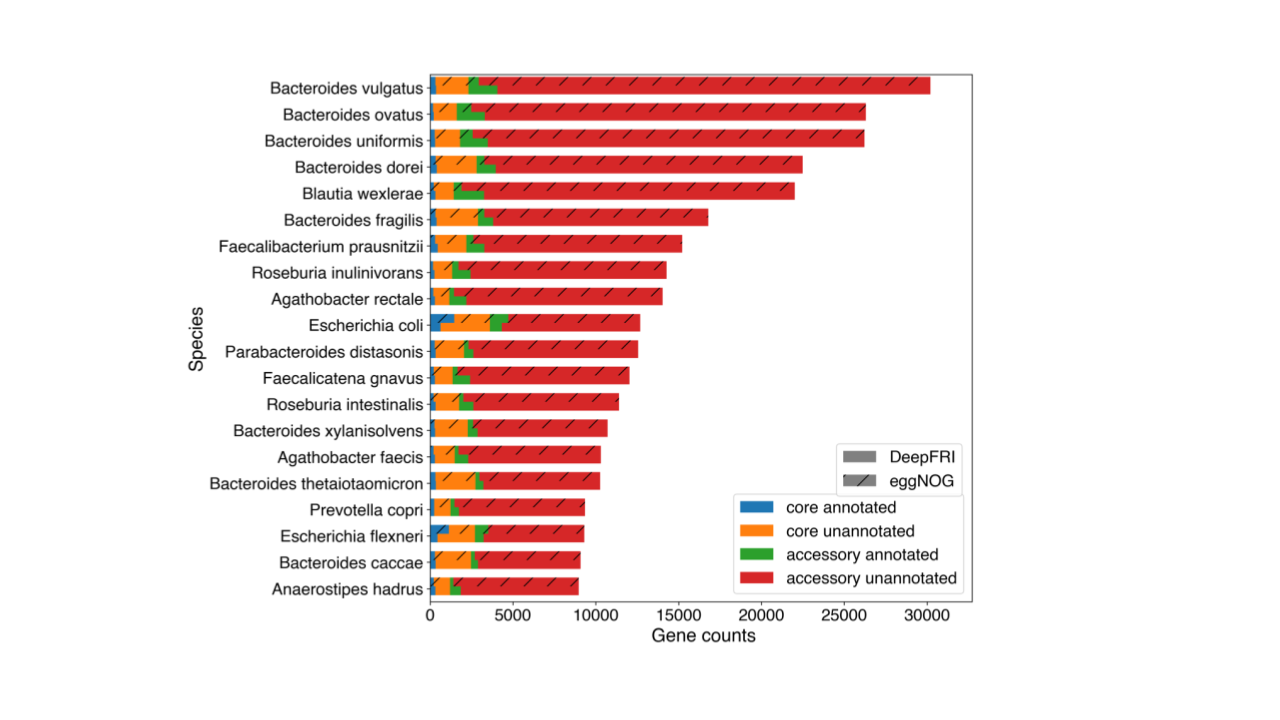

Supplement: FIG S8 [file msystems.01178-22-s0008.tif]

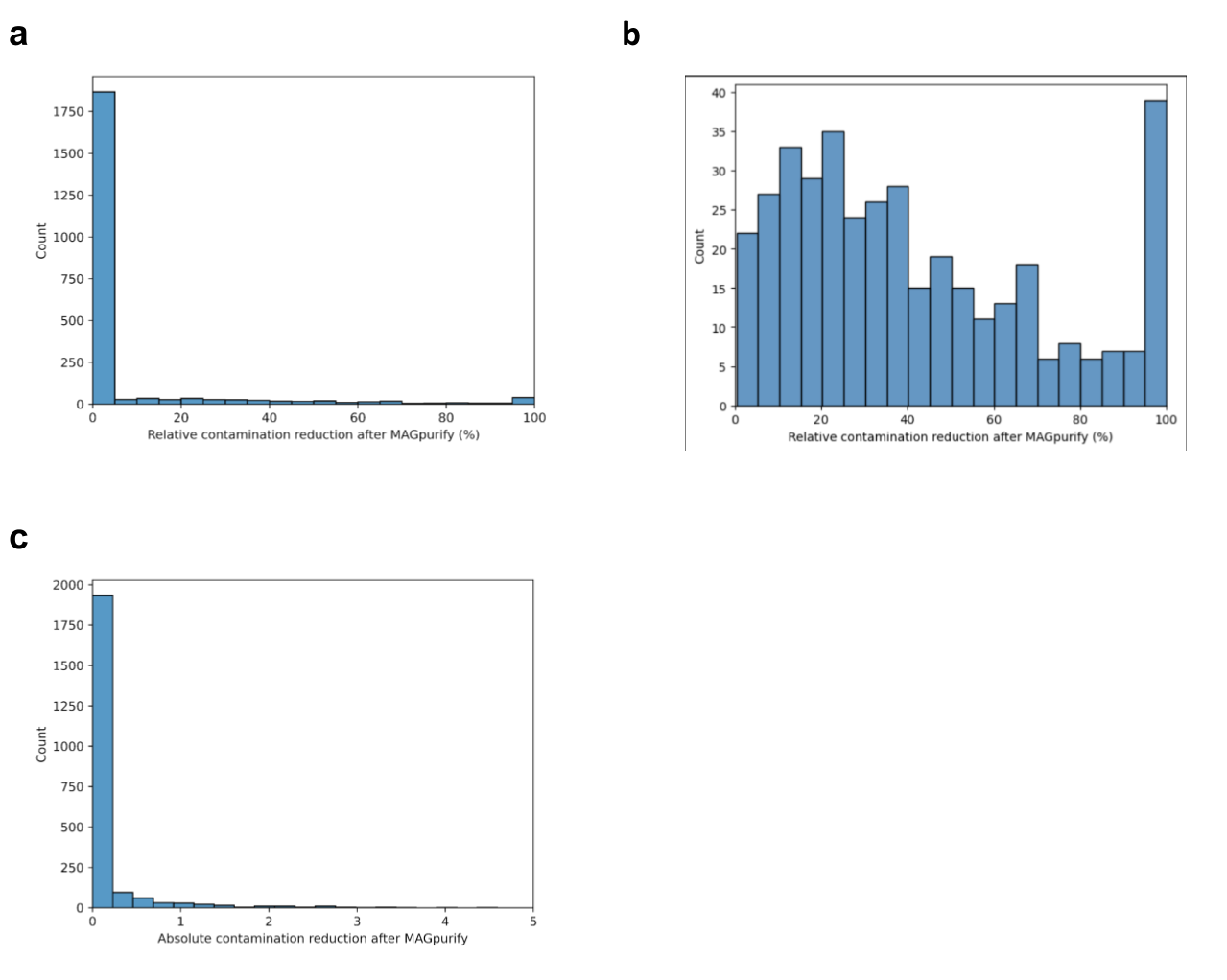

Supplement: FIG S9 [file msystems.01178-22-s0009.tif]
